# Monthly Summary

**Published:** 1873-06

**Authors:** 


### MONTHLY SUMMARY.

---

*Brain Stimulants.*—A prominent clergyman in a neighboring city writes us, that for many years he has been in the habit of limiting his use of tea and coffee, and his " occasional cigar " to the latter part of the week, and as he fancies, with the result of being able to compose with less effort than when he has either abstained entirely from their use, or when, as once or twice, he has indulged in them continuously for a brief period.

Herein is a valuable suggestion to brain workers in any profession, the exigencies of which call for occasionally increased and severe efforts. Tea, coffee, tobacco and alcohol, by retarding the changes in the tissues of the body, which is their physiological action, are supposed to allow the energy thus conserved to manifest itself in the higher form of cerebral activity—in simpler language, they are stimulants to the nervous system; and, in the proper dose, there can be no question that they do exalt and stimulate brain-action. But there is equally no question that the retarded tissue changes are at the expense of vitality generally—the vitality of the body; that is, its health and strength, being ever in relation to the newness of the atoms which compose the body—and these tissue-changes, the work of waste and repair, must be accelerated in some manner, and to a corresponding extent, in order to preserve the balance.

The obvious lesson to be gained from these facts is, that during periods of intense and unusual mental activity, a lawyer in trying an engrossing case, a banker during a financial stress, a company officer at periods of increased responsibility, an editor or political leader carrying through an important measure—that at such times brain-work may be done with more facility and at less expense, by a judicious use of this class of agents,—provided that the balance be struck at once when the necessity for them is obviated.

The means of restoring the balance include first, abstinence from the agents themselves; second, comparative rest for the brain; and lastly, and quite as importantly as the preceding, those measures which accelerate tissue-changes, and of which the essential ones are physical exercise and bathing—notably the Turkish bath—and nutritious, easily assimilated food, by the first of which, the breaking down of the older particles, and the excretion of poisonous waste-matter are facilitated, and so tissue-change in the interest of waste is promoted, while the last furnishes material for renewal and growth.

With such a regimen, based on an intelligent application of means to ends, we would have fewer cases of men prematurely breaking down under efforts they might make with ease, did they only know when and how to open the throttle-valve, or to put on the brakes. This view of the subject must not be construed into an argument for a mere sensual indulgence. It is intended for men as they are, and with regard to conditions as they exist. These agents are used, and probably always will be. They have their uses; and knowledge of these will do more to prevent than the wholesale condemnation which frequently arises from ignorance.—*Hygiene.*

---

*Labor.*—"Labor," says the Rev. Newman Hall, "as a mighty magician, walks forth into a region uninhabited and waste; he looks earnestly on the scene, so quiet in its desolation; then waving his wonder-working wand, those dreary valleys smile with golden harvests—those barren mountain slopes are clothed with foliage—the furnace blazes—the anvil rings—the busy wheels whirl round—the town appears—the mart of commerce, the hall of science, the temple of religon rear high their lofty fronts—a forest of masts, gay with varied penmons, rises from the harbor—the quays are crowded with commercial spoils, the peaceful spoils which enrich both him who receives and him who yields— representatives of far off regions make it their resort—science enlists the elements of earth and heaven in its service—art, awaking, clothes its strength with beauty—literature, new born, redoubles and perpetuates its praise—civilization smiles—liberty is glad—humanity rejoices—piety exults, for the voice of industry and gladness is heard on every hand; and who, contemplating such results, will deny that there is dignity in labor?"

*Controllng Sex in Butterflies*—A suggestive article as to the possibility of controlling sexes in butterfles has been communicated to *The American Naturalist* by Mrs. Mary Treat, and from the results of numerous experiments she finds occasion to believe that larvæ to which the freshest and most tempting food was supplied in unlimited quanity nearly always developed into female butterflies while those for which the supply of food was limited almost as uniformly proved to be males, Dr Packard is, however, inclined to think that the sex of this insect, as well as that of all animals from eggs, is determined at or about the time of conception, or at least, early in the embryonic conditions In the honey bee, especially, it has been proved that the sex is decided at the time the egg leaves the oviduct. The sex in man ,according to Koeliker, becomes fixed toward the end of the second month of œtal life.—*Scientific American.*

---

*A Sure Test as to Whether Life is Extinct or Not.*—A few years since a price was offered by the Paris Academy of Science for a sure and easily-applied test of the presence of death. In answer to this, Hugo Magnus, of Breslau, contributes a paper on this subject to Virchow's *Archives.* Dr. Magnus directs his attention to the vegetative phenomena, since among these may be found those peculiar to functlons which will bear being reduced to a minimum, but upon the stoppage of which death follows at once. Now there are two symptoms the functions of which are never completely suspended during life, viz : the circulatory and respiratory. Choosing the former of these, Dr, Magnus resorts to a very simple method, which is thus described :—

" If a limb of the body—a finger is best for the purpose—be constricted by a strong ligature quite tightly, there will be seen if the subject be yet alive, a reddening of the constricted member. First, the part in question becomes red, and the red color becomes darker and darker, and deeper in hue, till it is finally converted into a bluish-red, the whole being, from its tip to the ligature which encircles it, of a uniform color, except, that at the region immediately around the ligature itself there is to be seen a narrow ring, which is not bluish-red but white." On those whose skins are thickened and hard from work it may be necessary to choose some other part than a finger, as, for example, a toe or the tips of the ear. This evidence is as sure to be brought about in the living body as it is certain to be absent in the corpse. The bluish coloration of the finger-tips, so often observed in the dead body, need not be regarded as a source of fallacy, for after ligation of a finger, as long as life is present, the whole limb, from the point of ligation to the extremity, will be uniformly blue-red. Large limbs, on account of offering facilities for the flow of the venous blood through the deeper veins, do not serve as well as the small extremities, in which the tissues are more easily compressed against bone.—*Med. Record.*

---

*False Teeth.*—False teeth have their disadvantages as well as their advantages. They caused the death of Cuvier and the discomfiture of Lord Brougham. Cuvier, impatient at the interruptions of that perpetual interrupter, M. Glais-Bazoin, in the National Assembly, rose so impatiently to answer him, that he jerked his teeth on to the floor of the Assembly, and, stooping not less precipitately to pick them up, fell head foremost, and struck his head against the floor so heavily as to give rise to the illness which proved fatal to him. M. Glais-Bizoin, then a very young man, promised himself to abstain from his fatal habit of incessantly interrupting; but he was incorrigible. Lord Brougham, in the course of the proceedings of a great meeting of the Social Science Association, of which he was President, was stopped in the middle of a speech by his teeth falling out. After groping on the floor, and on presently resuming his speech, he made the best of the incident by observing that "our teeth are the source of troubles from infancy to old age."—*Brit. Med. Jour.*

---

*Dental Accidents During Anæsthesia.*—At the last meeting of the Pathological Society of Great Britain, Mr. F. Henry said he would like to make a few observations in respect to an incident which occurred a few days since in his operating room, and which suggested a probable cause for the unfortunate casualty at Exeter. A lady, desirous of having a lower molar tooth, very much decayed, removed under nitrous oxide, was placed under the influence of that agent. It was administered to her twice, and upon recovering after the second administration she exclaimed that she could not swallow and was choking. From these sensations she was relieved after one or two attempts at swallowing. Upon examining the tooth, it was found that two sides of the crown were missing. They were searched for, but could not be found. They had doubtless lodged somewhere in the throat, probably under the glottis. Viewing this case in connection with that at Exeter, he thought it most probable that in the latter the missing fragment of the prop lodged under the glottis and finally in the larynx.—*Med. & Surg. Reporter.*

---

Leprosy stills prevails to an alarming extent in the Sandwich Islands. The doctors can find no remedy. The lepers are isolated and live in large communities by themselves under rigid laws of exclusion from other mortals.

*Professor Haeckel on the Origin of Man.*—In an article in the *Scientfic American* of April 12, 1873, certain theories of Professor Haeckel, of Jena, are set forth. These to say the least, are very curious and apparently opposed to every principle of common sense.

In substance, the Professor says that man at the outset is an egg, a little cell the one hundredth of an inch in diameter, The egg becomes an embryo, brought about by the origiual cellule being divided into two, these two into four, etc., continually increasing in number until a quorum is formed, when they lay their heads together, so to speak, nominate committees to attend to particular duties. and finally, all agreeing, like good citizens, to abide by the laws (made by themselves?). set to work to form a perfect man or woman precisely like those who for thousands of years have been striving to obtain a living from the bosom of their mother earth. But stay, I am too hasty. We are to suppose that in case our present circumstances are ever so altered as to render it neccessary that man should become a monkey or a winged individual like the angels our forefathers believed in, then these tiny cellules will go to work accordingly and clothe us with hair or feathers, as the case may be.

What a beautiful theory! What wisdom these little cells display in thus cutting themselves up indiscriminately to build up the perfect man with his noble nature, his glorious ambition, his high attainments.

Can it be possible that any reasonsble person would entertain such a theory as the above? Must we be led to believe that particles of matter (themselves composed of elements well known to us) are capable of acting on concert in their own responsibility, as it were, like ourselves and so creating man to suit the circumstances, as we would construct a goverment or a machine for certain ends or purposes? Granting even this to be so, then we must go back to the elements of which the embryo itself is formed. By what power are these brought into the form of an egg or cellule? Since the same elements combine to form man and monkey, trees and plants, surely we must look for the orignal reasoning power in them, if we discard the idea of a Supreme Creator, and not in any particles or cellules formed by them. Thus we have oxygen, hydrogen, nitrogen and carbon transformed into reasoning beings, who either created themselves or have existed through eternity, and have the power of creating at pleasure man, beast or plant from their own substance

Here we see that certain of mankind, in their frantic endeavors to ovethrow the claims of theology and the distasteful idea of a Supremee Being, with power to reward or punish, unconsciously set up a god formed of the elements, who has at least the power to create and destroy at will; for we must not lose sight of the fact that to plan and accomplish a given purpose requires an effort of will, be it natural or supernatural, and that there is a vast difference between the controlling influence of mind and the passive obedience of matter to established laws; the existence of which laws itself implies the prior existence of a law making power.

I would suggest that the learned Professor turn his attention to the moral of an old and well known fable, for his great mountain of words, with monstrous labor, brings forth an exceedingly microscopical mouse of proof for Mr. Darwin, who will surely exclaim "save me from my friends."—*Scientific American.*

J. L., Washigton, D, C.

---

*Dangers of Chromic Acid.*—Mr. Gubler remarks in the *Edinburgh Medical Journal*, that chromic acid is one of the most powerful of caustics. Only the monohydrous sulphuric acid at all approaches it in strength. It acts rapidly, setting free a considerable amount of heat, so that the temperature may rise to 125 or 150 degrees. If we plunge a small animal, such as a mouse, into a concentrated solution of chromic acid, it is instantly reduced to a cinder; and the ebullition is so great that unless care be taken, the mouse and a part of the solution are forcibly ejected.

This caustic applied over an extensive surface may therefore give rise to a deep slough. Further, the absorption of chromic acid is not free from danger, and patients have been poisoned by a too extensive application of this caustic poison to the surface of their bodies.—*Med. & Surg. Reporter.*

---

*Glycerine Lotion.*—For softening the skin of the face and hands, especially during the commencement of cold weather, and also for allaying the irritation caused by the razor:

Triturate 4½ grains of cochineal with 1½ fluid ounces of boiling water, added gradually; then add 2½ fluid ounces of alcohol. Also make an emulsion of 8 drops of attar of roses with 30 grains of gum Arabic and eight fluid ounces of water; then add three fluid ounces of glycerine and 10 fluid drachms of quince mucilage. Mix the two liquids.—*Zeitschrift, etc.*, 1873.—*The Southern Med. Record.*

---

It is stated that a little girl in Philadelphia died a few days ago from hydrophobia taken by biting off a thread after mending a rent in her dress which her dog had torn in play. She had the disease in its most frightful and distressing form. This is probably a case of idiopathic tetanus or lock jaw. the symptoms of which often simulate to some extent those of hydrophobia.

*The Wonders of the Deep.*—During the recent passage of the British exploring ship Challenger from England to tbe West Indies, the sounding line and dredge were kept constantly going. The former showed that a pretty level bottom runs off from the African coast, deepening gradually to a depth of 3,125 fathoms at about one third of the way across to the West Indies. If the Alps, Mont Blanc and all, were submerged at this spot, there would still be half a Mile of water above them. Five hundred miles farther west there is a comparatively shallow part a little less than two miles in depth. The water then deepens again to three miles, which continues close over to the West Indies. At the deepest spots both on the east and west side of the Atlantic, the dredge brought up a quantity of dark red clay, which contained just sufficient animal life to prove that life exists at all depths. No difficulty was experienced in obtaining these deep sea dredgings, and it was merely a question of patience, each haul occupying twelve hours. In depths over two miles little has been found, but that little was totally new. One of the lions of the cruise is a new species of lobster perfectly transparent. Not content with obtaining animals with eyes so fully developed that the body may be said to be an appendage, a new crustacean has now been dredged up, in which the body has cut itself clear of the eyes altogether, and the animal is totally blind. It has no eyes, or even the trace of an eye. To make up for its deficiency Nature has supplied it with the most beautifully developed, delicate lady like claws, if one may use the term, it is possible to conceive. Nearer the West Indies, in a depth of only half a mile, some similar creatures were brought up, and here the claws, longer than the body, are armed throughout with a multitude of spike-like teeth, looking more like a crocodile's jaw than anything else. At a short distance from Teneriffe, in a depth of a mile and a half, a rich and extremely interesting haul of sponges and coral was obtained, but the latter was unfortunately dead.—*Scientific American.*

---

The smallest known race is that of the bushman of Southern Africa, the largest that of the Patagonian of South America. The mean hight of the bushman is four feet three and a half inches, and that of the Patagonian five feet eight inches.

---

*Neuralgic Pill.* Brown Sequard.– ℞. Ext. belladon., one-sixth of a grain; ext. stramon., one-fifth of a grain; ext. cannab. ind., one-fourth of a grain; ext. aconit., one-third of a grain; ext. opii, one-fourth of a grain; ext. hyosciami, two-thirds of a grain; ext. conii, 1 grain. For one pill.—*Detroit Review of Med. & Phar.*

